# Medical dispatchers’ perception of visual information in real out-of-hospital cardiac arrest: a qualitative interview study

**DOI:** 10.1186/s13049-018-0584-0

**Published:** 2019-01-25

**Authors:** Gitte Linderoth, Thea Palsgaard Møller, Fredrik Folke, Freddy K. Lippert, Doris Østergaard

**Affiliations:** 1 Emergency Medical Services Copenhagen, University of Copenhagen, Telegrafvej 5, DK-2750 Copenhagen, Denmark; 2 Department of Anaesthesia and Intensive Care, Bispebjerg and Frederiksberg Hospital, University of Copenhagen, Bispebjerg Bakke 23, DK-2400 Copenhagen, NV Denmark; 3 Copenhagen Academy for Medical Education and Simulation, University of Copenhagen, Herlev Ringvej 75, DK-2730 Herlev, Denmark

## Background

Dispatcher-assisted cardiopulmonary resuscitation (DA-CPR) is highlighted in 2015 resuscitation guidelines [1] because instructions improve bystander CPR rates [2–4], reduce the time to CPR [4–6], increase the number of chest compressions delivered, and might improve patient outcomes following OHCA [7, 8]. However, DA-CPR can be difficult because the dispatchers are placed in a complex, nonvisual environment, guiding bystanders who are in a stressful situation and often have limited basic life support experience. Simulation studies have shown that video transmitted from bystanders’ smartphones can be a valuable tool for dispatchers to facilitate DA-CPR [9] and that it can improve the lay rescuers’ confidence [10].

Medical dispatchers have also indicated that it could be a benefit to receive visual information based on simulations of OHCA scenarios [11]. This is in agreement with our previous study in which we used closed-circuit television (CCTV) recordings from OHCA, which indicated that the dispatchers’ situation awareness was challenged by not being able to see the victim as well as the bystanders’ reactions, though the medical dispatchers were not interviewed specifically about their perception of what happened on scene [12]. Therefore, medical dispatchers’ understanding of a real OHCA scenario and their reflections about receiving visual information in real-life cardiac arrest situations is unknown. We hypothesised that it could be a benefit for dispatchers to perceive visual information from the scene of OHCA.

The aim of the present study was to explore the medical dispatchers’ perception of the bystanders’ responses and dispatchers’ reflections about the added value of visual information in real out-of-hospital cardiac arrest (OHCA) situations investigated with CCTV recordings.

## Method

### Setting

The Emergency Medical Dispatch Centre (EMDC), Copenhagen, Denmark serves 1.8 million inhabitants. All 112 emergency calls are initially handled by a call centre that identifies the location and then forwards all medical calls to an EMDC. An emergency medical dispatcher answers the call and determines the appropriate response, while a technical dispatcher handles the logistics of simultaneously dispatching ambulances. The medical dispatchers are specially trained registered nurses and paramedics. The decision-making process is supported by a standardised national criteria-based dispatch tool (Danish Index for Emergency Care) [13]. In case of OHCA, the medical dispatchers guide the bystanders to perform CPR until arrival of the ambulance and to localise and use the nearest automated external defibrillator (AED).

### Study design

**Fig. 1:**
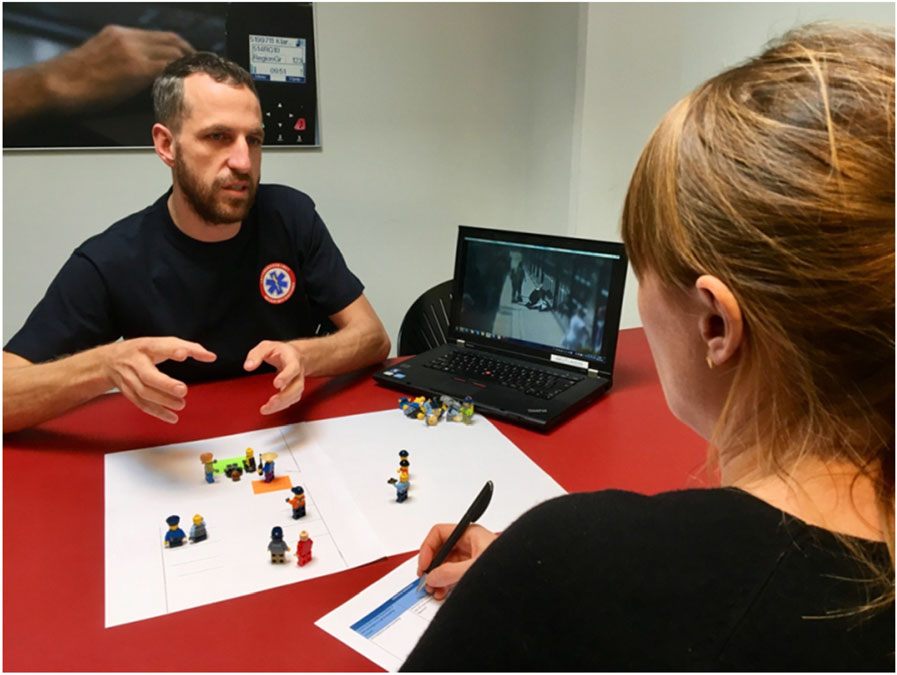
Illustration of medical dispatcher showing bystander response using minifigures to allow visualisation of his own perception of the situation

The study was a qualitative study designed as an explorative interview study including medical dispatchers who had previously handled a case of OHCA captured on CCTV. The CCTV recordings were collected in relation to a previous study (11). The primary researcher invited by e-mailed 10 out of 21 relevant medical dispatchers to participate. We chose medical dispatchers who were available (still employed and not sick or on maternity leave) and where the CCTV footage of the OHCA showed most information about the victim and the resuscitation attempt. No relationship to the medical dispatchers were established before the study. The medical dispatchers were interviewed individually at the EMDC with a timeframe from 6 months to one year after collection of the CCTV recordings. The duration of the interview was approximately one hour. First, the medical dispatcher listened to the audio recording until a natural break occurred in the conversation between the caller and the medical dispatcher – for example, when the medical dispatcher had to register information in the electronic system or changed the subject. The interviewer then asked the medical dispatcher to visualise his or her perception of the situation using mini-mannequins (Fig. 1). The medical dispatcher listened to the remaining part of the audio recording, thereafter the interview was continued. Finally, the medical dispatcher watched the CCTV, listening to the audio recording of the emergency call simultaneously, and was then interviewed about his/her understanding of the OHCA-scenario and reflections on receiving visual information.

An interview guide for the semistructured interviews was developed prior to the study (Additional file 1: Appendix). A pilot interview was conducted with a medical dispatcher to evaluate the interview guide and the setup. The first (GL) and last authors (DOE) participated in the first three interviews. Thereafter, the first author conducted the interviews. Nobody else was present at the interviews. The interviews were videotaped, and the audio files were transcribed verbatim.

### Analysis

We applied a qualitative research methodology using thematic analysis with an explorative and inductive approach. The first author followed Braun and Clarke’s six-step analytical approach, which has the following steps: familiarising with data, generating initial codes, searching for themes, reviewing themes, defining and naming themes, and producing the report [14]. Codes and themes were organised using Nvivo10 software (QSR International Pty Ltd., Australia). A single episode of great importance was sufficient to form a theme. The team of researchers analysing the data consisted of two physicians with some experience in qualitative research (GL and TPM) and a professor in medical education who also had experience with qualitative research (DOE). All participated in all steps of the analytic process. After analyzing the eight interviews data saturation was research, but as the last interviews had been planned – these were conducted as well. Based on our previous analysis of the CCTV recordings we had a preconception that the CCTV would be a benefit that could affect our analysis. The themes were therefore presented to the medical dispatchers for any comments; none were given.

## Results

Ten medical dispatchers were interviewed. Five of these were women. Four were registered nurses, five were paramedics, and one was a paramedic trainee. The length of experience in emergency medical dispatching was 28 months (21–45 months), median (range). None of the contacted medical dispatchers declined to participate. The characteristics of the situations in the 10 CCTV recordings are shown in Table 1.

**Table 1:** Characteristics of the out-of-hospital cardiac arrest cases (*n* = 10)

	CCTV recordings
Location	
Train or train station	4
On the pavement outside a shop	3
Airport	1
Bowling centre	1
Convention centre	1
Bystander/Caller^a^	
Number of bystanders participating in CPR, retrieving an AED, guiding ambulance personnel to the victim, calling for health care professionals, or having the conversation with 112	
2 bystanders	4
3 bystanders	3
> = 4 bystanders	3
Caller’s relationship to patient	
Stranger	10

^a^The “caller” was the person who primarily had the conversation with the emergency medical dispatcher

### Improved understanding of the cardiac arrest scenario

The medical dispatchers expressed an improved understanding of the OHCA scenario after they watched the CCTV recording. The CCTV footage provided more information about the victim, the physical setting and the bystander response. Table 2 shows themes, subthemes, and data extract examples.

**Table 2:** Themes related to the medical dispatchers’ perceived benefits with adding visual information from closed-circuit television (CCTV) to handling of out-of-hospital cardiac arrests (OHCAs)

Themes and subthemes	Data excerpts/examples and medical dispatchers’ quotes
*Improved understanding of the OHCA scenario*	
The victims’ condition and position	- “*You would be able to see if he is breathing or has any movements and other things*.” - “*He is unable to breathe when he is held like that*.”(…) *“I thought he was lying down*.” (…) *-* “*Many bystanders say: ‘I cannot tell. I do not know.’ It would be a benefit to get your own eyes on the scene.”* (The comment refers to bystanders’ descriptions of the patient’s condition).
The physical room/location	*-“I have no idea of the setting. If I had known that he was lying in the doorway, I would have regimented that they pulled him away from the doorway, so they could get some space for working*.” (…) “*First I thought it was at the reception in another building* (…). *The ambulance drives to the wrong building*.”
Bystanders’ response	
-Bystanders helping/present	- “*In this case two more persons are present without the caller mentioning them*.”
-CPR performed	*-* “*Only one person did CPR. It surprises me. That was not what I thought*.” - “*If the CPR should have been correct, the rate should have been faster.”* - “*I do not really know if they do compressions on thorax or in the stomach. I would be able to see that with these pictures*.”
-Callers’ position	- “*If I could have seen that caller was so far away from the victim* (…). *I would have asked caller to get closer to the victim*. (…) *The information I get is almost useless*.”
*Potential benefits for dispatcher-assisted CPR*	
Improved communication	- “*It would be great to have visual contact*. (…) *It can be so difficult to know from just listening. Is what I ask about perceived correct*? (…) *Normally we use our eyes a lot when working in the field*.” - “*when you are not present, you do not know what is really going on unless you make the bystanders explain and confirm all the time. Are you doing this and that*? *You have to ask all the time*.”
Improved guidance of bystanders/Dividing tasks among bystanders	- “*I would be better to* guide [with CCTV]*, because I can see what is really going on and what is not happening*.” (…) “*You can see if the bystanders do what you tell them to*.” - “*You can give more direct guidance when you do not use time trying to understand what is going on*.” (…) “*I could have seen that man. I was not aware of his presence. I could have said, ‘ask the man to help get the victim on the floor*.’”
Quality CPR	- “*The CPR quality could improve, because you could say* ‘*he has to press harder*,’ *or* ‘*now the woman who is just standing there has to take over*.’” - “*We need feedback all the time. For compression it is like, ‘Now! Now! Now!’ on the phone. We only get an instant image on the phone. We do not know the real rhythm*.”

In cases where the CCTV contributed with more close-up pictures of the victim, the dispatchers were able to observe the victim’s condition including consciousness, skin color, and respiration patterns. In all cases, the dispatcher could observe when the victim stopped having voluntary movements, as an indicator of unconsciousness. Furthermore, the dispatcher received information about the victim’s position: if the victim was held in sitting position, lying on the back, or lying on the side. The dispatchers sometimes thought the victim was sitting independently when he or she was actually being held in the sitting position, because the caller stated that the victim was “sitting.” In one situation, the dispatcher thought the victim was sitting by himself and therefore did not identify OHCA.

The dispatcher also perceived more information about the physical setting with the CCTV recording, including the location of the victim and hence, the bystanders’ working conditions. In one case, the victim was lying in a doorway, which made it difficult for the bystanders to assist the victim. The dispatcher was not aware of this before he watched the CCTV recording.

All OHCAs were in public places. If the caller did not mention the number of bystanders directly, all medical dispatchers thought there were several bystanders in the surroundings when the OHCA happened in daytime. The medical dispatchers’ perceptions sometimes failed when it came to estimating the number of bystanders helping the victim or performing CPR. Often the dispatchers thought more bystanders were involved.

The medical dispatchers often presumed that the quality of CPR performed was adequate, and they asked few or no questions to get information about this, so in case of inadequate bystander CPR, the medical dispatchers’ perceptions were not in accordance with reality.

The CCTV recordings provided an improved understanding of how the CPR was performed, including shift among laypersons to provide compressions, hands-of-time, hand position, compression rate, and continuous evaluation. Furthermore, most medical dispatchers assumed that the callers were very close to the victims. In many cases, however, the caller was standing some distance from the victim.

### Potential benefits for dispatcher-assisted CPR

The medical dispatchers expressed that their improved perception of the OHCA scenario could improve the communication with the bystanders, their ability to guide more bystanders and the quality of CPR in the future. (Table 2 shows themes, subthemes and data extract examples).

The communication could be improved because it would be easier for dispatchers to judge whether their messages were understood and actions initiated by the caller. Some dispatchers also thought time could be saved in gathering information, because the amount of necessary questions could be reduced. The dispatcher was also less dependent on continuous information from the caller and could potentially respond faster to changes.

The medical dispatchers expressed that the improved understanding of the OHCA scenario would make it possible to participate more actively and make it easier to guide the bystanders. The bystanders who did not assist were seen as potential resources by the medical dispatchers, who.

suggested better involvement of these bystanders at the scene. The dispatchers believed that CCTV provided an opportunity to correct poor CPR quality and continuously evaluate the CPR.

### Challenges

Table 3 lists the themes, subthemes, data extract examples related to challenges of CCTV. The medical dispatchers also expressed concerns regarding the use of CCTV, including receiving too much information, which might be confusing when they had to alternate between several points of focus. Furthermore, concerns were expressed about potentially delayed dispatch of an ambulance because the dispatcher might use time analysing the pictures before dispatching the ambulance. Potential logistic and technical issues were also raised, such as time spent on connection for live transmission and the quality of the pictures. Furthermore, one dispatcher had thoughts about psychological aspects for the dispatcher in watching these recordings. This was the only theme that only contained one episode.

**Table 3:** Themes related to the medical dispatchers’ expressed challenges with adding visual information from closed-circuit television (CCTV) to handling of out-of-hospital cardiac arrests (OHCAs)

Themes for challenges	Data excerpts/examples and medical dispatchers’ quotes
Several points of focus	- “*If my eyes do not catch the victim immediately on the CCTV, my attention could be drawn away from important information on the phone*.”
Delayed dispatch	- “*You have to dispatch the ambulance fast. It could be a problem if you would analyze the pictures instead of just believing the caller*.”
Logistics and poor quality of the pictures	- “*The video connection has to be right away, and the picture has to be good*.”
Psychological aspects	- “*You have to be prepared. They could be tough pictures to watch*.”

## Discussion

The medical dispatchers expressed that the CCTV footage contributed valuable information about the OHCA patient, the physical setting, and the bystanders’ response. The improved understanding of the OHCA scenario with CCTV could improve DA-CPR because a dispatcher could more easily follow up on what really happens at the scene and provide better guidance to the laypersons participating in the resuscitation attempt. Challenges of receiving visual information could be logistics, delayed EMS dispatch, poor video quality, receiving too much information, which might be confusing when dispatchers have to alternate between several points of focus.

Our study is unique because it provided the dispatchers the possibility to see what really happened in an OHCA scenario and afterwards reflect on the use of visual information.

In our previous study, where we analysed CCTV recordings, we hypothesised that the medical dispatchers´ situation awareness and communication could be challenged by not being able to see the OHCA scenario [12]. A limitation of the study was that the medical dispatchers were never interviewed about their understanding of the OHCA scenario or their reflections on the added value of receiving visual information, as we did in the present study. The medical dispatchers expressed that visual information could improve their understanding of the OHCA scenario.

Our findings are in agreement with a study from Johnson and Bolle [11], where they conducted interviews with six dispatchers after the dispatchers’ participation in simulated cardiac arrest scenarios using video calls in five scenarios and normal audio calls in five scenarios. They found that video calls were more useful for obtaining information and providing adequate functionality to support CPR assistance compared to audio calls.

The only challenge with video calls was the risk of “noise,” because the video call provided more information and more impressions, some of which might be unnecessary. This was also in agreement with our study where the dispatchers questioned whether there could be “too much” information to handle. While visual information might improve dispatchers’ understanding of the rescuers’ situation and the assistance they provide, training in handling additional visual information during the emergency call seems necessary.

Other simulation studies have focused on CPR quality with video-instructed DA-CPR [15–20]. Lin et al. made a meta-analysis comparing video-instructed DA-CPR with audio-instructed DA-CPR and found that the compression rates were closer to the recommendations [9]. Improved hand position, correct depth, and correct ventilation rates also tended to be associated with video-instructed DA-CPR, but data were insufficient for meta-analysis. Time without chest compression (“hands-off time”) was not included in the meta-analysis, but this too might be shorter [20]. One limitation with video-instructed DA-CPR was that the time to first compression was longer [16]. In the studies, video-instructed DA-CPR started with an instruction of CPR via video transmission. In countries such as Denmark where CPR rates are high [21], and where basic life support (BLS) is mandatory in schools and when acquiring a driver’s license [22], it might be unnecessary to show CPR instruction to get CPR started. Focus could be on evaluating the CPR quality by giving the bystanders feedback. High-quality CPR remains essential to improving outcomes [23, 24]. The existing feedback on compression technique includes voice prompts, metronomes, visual dials, numerical displays, waveforms, verbal prompts, and visual alarms. However, no studies have yet demonstrated improved survival. The ERC guidelines 2015 [4] concluded that the use of CPR feedback or prompt devices during CPR should be considered as part of a broader system of care that should include comprehensive CPR quality improvement initiatives. The present study supports that visual contact might enhance dispatchers´ ability to give bystanders better feedback on CPR quality in the future.

The dispatchers in this study acknowledged that they could have asked for more relevant information to get a better perception of the situation. Visual information might make it possible for the dispatcher to ask focused questions in a situation with limited time and to lead and guide the bystanders accordingly. One can speculate on whether or not BLS courses also should include topics such as communication with the dispatchers – how to provide important information from the caller to the dispatcher, and how to use all available resources at the scene and follow guidance from the dispatcher. Real-life CCTV recordings could be used to prepare laypersons in handling OHCA. Furthermore, contact and supervision through video calls could improve the rescuers’ confidence in stressful emergencies [10].

### Limitations

This study has some limitations. The retrospective setting might influence the dispatcher’s perception, as the medical dispatchers may have acquired significant additional knowledge about the OHCA scenario content after handling the scenario. All dispatchers in the study knew that the case was about OHCA, but not all could remember the specific case. However, as the emergency call conversation proceeded, they received more information, which affected their perception at a given time. We tried to minimise this by introducing a break in the emergency call conversation.

The long time from the OHCA to the interview might be an advantage as none of the dispatchers could remember the OHCA case in detail. If the period is short, the dispatchers might remember the full OHCA scenario from start to end. This might affect their perception in the beginning of the call. On the other hand, the long duration could be seen as a limitation as their perception could be different from when they actually handled the call. The methodology of using available CCTV implies that the cases are not representative for all OHCA.

### Future perspectives

The number of CCTV cameras is limited and access might be restricted for medical dispatch centres. Smart phone ownership rates are high [25], and as the technology improves, citizens might expect video as part of an emergency call. Video call too EMDC is possible in some places in Asia (e.g., Taiwan, Singapore and South Korea), London and New Zealand but the usage has not been systematic investigated.

CCTV often gives a bird’s-eye view unless the right conditions for zoom are present, whereas smartphones provide a better view of the victim, including assessment of ventilation and agonal breathing, which is a well-known challenge in the identification of OHCA [26, 27].

The present study supports that visual contact might enhance dispatchers´ ability to give bystanders better feedback on CPR quality in the future. Next step must be to investigate if video call can improve the quality of CPR in real life and improve survival. However, before live transmission is introduced, issues such as logistics at EMDC and training of the medical dispatchers must be addressed [28]. The dispatchers need training on how to interact with the bystanders and guide the team. However, in simulations studies dispatchers found video-calls surprisingly easy to use and were surprised that they so quickly had adapted reliance on the picture [11].

## Conclusion

In conclusion, providing medical dispatchers with visual information from the location of OHCA might improve their understanding of the OHCA-scenario, which might enhance communication, their ability to guide more bystanders and improve the quality of cardiopulmonary resuscitation. Challenges with receiving visual information might be logistics, delayed dispatch, poor quality of the pictures and receiving too much information, which might be confusing when they had to alternate between several points of focus.

## Additional file

Additional file 1: **Appendix.** Interview guide to facilitate the interviews of the medical dispatchers who have handled an emergency call in case of an out-of-hospital cardiac arrest, which has been captured on closed-circuit television (CCTV). (DOCX 15 kb)
