# Supplementary material for: Medical dispatchers’ perception of visual information in real out-of-hospital cardiac arrest: a qualitative interview study
Source: Scand J Trauma Resusc Emerg Med. 2019 Jan 25;27:8. doi: 10.1186/s13049-018-0584-0 (PMC6347804; doi:10.1186/s13049-018-0584-0)
Supplement: Supplementary file 1 — Appendix. Interview guide to facilitate the interviews of the medical dispatchers who have handled an emergency call in case of an out-of-hospital cardiac arrest, which has been captured on closed-circuit television (CCTV). (DOCX 15 kb) [file 13049_2018_584_MOESM1_ESM.docx]

**Appendix 1.** Interview guide to facilitate the interviews of the medical dispatchers who have handled an emergency call in case of an out-of-hospital cardiac arrest, which has been captured on closed-circuit television (CCTV).

|  | **Interview questions** | **Supportive questions** |
| --- | --- | --- |
| Listen to the audio recording of the emergency call (including break) |  |  |
| Perception/understanding | Describe the scenario with the use of minifigures? | Based on the audio recording, could you illustrate your perception of the scene?  Where is the caller in relation to the victim?  Did the caller participate in CPR?  How many bystanders are present?  Did the bystanders have basic life skills? |
|  | What are your thoughts about the CPR performance? | Is the CPR quality good? |
| Watch the CCTV recording combined with the emergency call |  |  |
| Perception/understanding | What are your thoughts after seeing the CCTV? | Does anything surprise you?  Would you have had more information earlier if you had had CCTV? |
| Task management/reflection | Would you have done something different if you had had CCTV?  Would your assistance in CPR been different? | How could you have used CCTV in relation to CPR?  Did you use all resources present? |
| Future perspectives |  |  |
|  | Would the dispatcher benefit from visual contact during the emergency call? | Describe the benefits and/or drawbacks? |
